# Prediction of insect pest distribution as influenced by elevation: Combining field observations and temperature-dependent development models for the coffee stink bug, *Antestiopsis thunbergii* (Gmelin)

**DOI:** 10.1371/journal.pone.0199569

**Published:** 2018-06-22

**Authors:** Abdelmutalab G. A. Azrag, Christian W. W. Pirk, Abdullahi A. Yusuf, Fabrice Pinard, Saliou Niassy, Gladys Mosomtai, Régis Babin

**Affiliations:** 1 International Centre of Insect Physiology and Ecology, Nairobi, Kenya; 2 Department of Zoology and Entomology, University of Pretoria, Pretoria, South Africa; 3 Department of Crop Protection, Faculty of Agricultural Sciences, University of Gezira, Wad Medani, Sudan; 4 UPR Bioagresseurs, Centre de Coopération Internationale en Recherche Agronomique pour le Développement, Nairobi, Kenya; 5 Bioagresseurs, Université de Montpellier, Centre de Coopération Internationale en Recherche Agronomique pour le Développement, Montpellier, France; US Department of Agriculture, UNITED STATES

## Abstract

The antestia bug, *Antestiopsis thunbergii* (Gmelin 1790) is a major pest of Arabica coffee in Africa. The bug prefers coffee at the highest elevations, contrary to other major pests. The objectives of this study were to describe the relationship between *A*. *thunbergii* populations and elevation, to elucidate this relationship using our knowledge of the pest thermal biology and to predict the pest distribution under climate warming. *Antestiopsis thunbergii* population density was assessed in 24 coffee farms located along a transect delimited across an elevation gradient in the range 1000–1700 m asl, on Mt. Kilimanjaro, Tanzania. Density was assessed for three different climatic seasons, the cool dry season in June 2014 and 2015, the short rainy season in October 2014 and the warm dry season in January 2015. The pest distribution was predicted over the same transect using three risk indices: the establishment risk index (ERI), the generation index (GI) and the activity index (AI). These indices were computed using simulated life table parameters obtained from temperature-dependent development models and temperature data from 1) field records using data loggers deployed over the transect and 2) predictions for year 2055 extracted from AFRICLIM database. The observed population density was the highest during the cool dry season and increased significantly with increasing elevation. For current temperature, the ERI increased with an increase in elevation and was therefore distributed similarly to observed populations, contrary to the other indices. This result suggests that immature stage susceptibility to extreme temperatures was a key factor of population distribution as impacted by elevation. In the future, distribution of the risk indices globally indicated a decrease of the risk at low elevation and an increase of the risk at the highest elevations. Based on these results, we concluded with recommendations to mitigate the risk of *A*. *thunbergii* infestation.

## Introduction

The antestia bug, *Antestiopsis thunbergii* (Gmelin 1790) (Hemiptera: Pentatomidae) is one of the major insect pests of Arabica coffee in eastern and southern Africa [1, 2]. The pest feeds on coffee leaves, buds, flowers and berries leading to direct damage [3]. By feeding on immature berries, the bug causes premature fruit fall and necrosis of the beans [4]. In addition, feeding lesions allow the fungi *Nematospora* spp. to colonise the beans, leading to endosperm rotting and a damage known as “zebra beans” [5]. *Antestiopsis thunbergii* is also supposedly involved in the transmission of a bacteria *Pantoea coffeiphila* (Enterobacteriaceae), causing a flavour defect of coffee beverage known as ‘potato taste’ defect (PTD), which threatens coffee production in the African Great Lakes region [6, 7]. The economic threshold for taking action against this pest is as low as one or two bugs per coffee tree on average, depending on the level of damage found in the country [4, 8, 9]. Contrary to most other coffee pests like the coffee berry borer, *Hypothenemus hampei* Ferrari, *A*. *thunbergii* thrives in high elevation Arabica coffee. The pest is present in plantations between 1000 and 2100 m asl, but with a strong preference for plantations of the highest elevations [2]. Two closely related antestia bug species, *A*. *intricata* (Ghesquière and Carayon) and *A*. *facetoides* Greathead, are more common in coffee plantations at low-medium elevation (1000–1600 m asl) [1, 2, 10]. A recent study by Azrag et al. [11] suggested that *A*. *thunbergii* preference for higher elevations might be due to the pest’s adaptation to cool habitats in the tropics.

Temperature is the most important environmental factor that affects insect distribution [12, 13], and it is highly correlated with elevation [14]. Variation in temperature affects insect population dynamics through insect physiology and behaviour [15]. Indirect effects also are expected due to the impact of temperature on host plants and natural enemies [12]. A recent study by Azrag et al. [11] provided an insight into *A*. *thunbergii* thermal requirements using temperature-based development models. These models showed that the pest was able to survive and develop under a temperature range of 14.6–32.9°C. However, based on simulated life table parameters, growth of the rearing population was restricted to a temperature range of 19–25°C [11]. Such thermal requirements suggest an adaptation to temperatures cooler than expected for a tropical insect.

In tropical and temperate regions, change in distribution and abundance are expected for many insect pests as a result of global warming [12, 16, 17]. For example, in east Africa, *H*. *hampei* is currently an issue for coffee production at elevation of up to 1500 m asl. By the year 2050, rising temperatures may lead to higher populations on Arabica coffee at elevations up to 1800 m asl, leading to reduction in both production and quality of renowned Arabica coffees [16, 18]. In contrast, the abundance of some herbivorous insects is expected to decrease due to their vulnerability to high temperatures [12]; yet, such scenarios have not been well documented.

Considering previous findings by Azrag et al. [11], it becomes feasible and necessary to predict the risk of *A*. *thunbergii* infestation in coffee of the east African highlands. Therefore, the general objectives of the present study were first to elucidate the relationship between *A*. *thunbergii* current distribution and elevation, and second to assess the potential impact of temperature change on the future distribution of the pest. To achieve these objectives, we first analysed this relationship using the pest abundance surveyed along an elevation gradient. In a second step, we used simulated life table parameters, incorporating the full life history of the pest, obtained from temperature-dependent development models [11] to generate risk indices based on temperature, using Insect Life Cycle Modelling software (ILCYM) [19]. The indices were calculated and mapped on the same elevation transect using temperature data collected in the field, in order to compare index distribution to this obtained from the field survey. Finally, to predict the potential impact of temperature increase on the future distribution of *A*. *thunbergii*, we generated and mapped the risk indices on the transect with simulated temperature data for the year 2055 from the Representative Concentration Pathway warming scenario 4.5 (RCP 4.5).

## Materials and methods

### Study site

**Fig 1:**
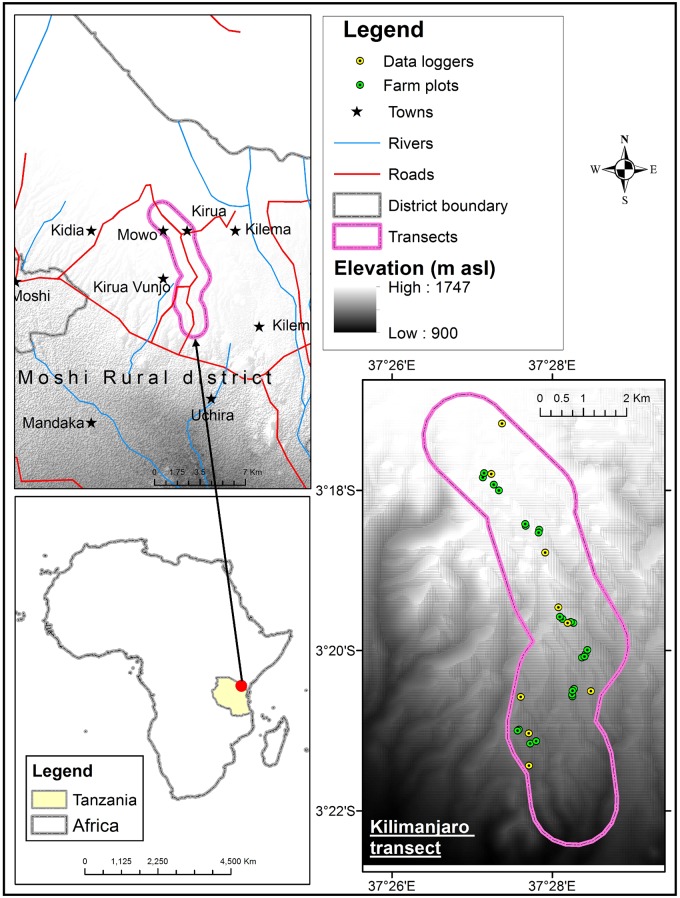
Location of the study transect over an elevation gradient on the south-eastern slope of Mount Kilimanjaro, in Moshi district, Tanzania. The transect delimited in pink is approximately 11 km-long and 2 km-wide with a total surface area of around 22.2 km^2^.

Our study targeted the Arabica coffee growing area of south-eastern slope of Mount Kilimanjaro in Tanzania (S3.28° E37.43° and S3.47° E37.50°). A transect of approximately 11 km-long and 2 km-wide with a total surface area of around 22.2 km^2^ was delimited over an elevation gradient of 1000–1700 m asl (Fig 1). This agricultural area is characterised by a bimodal rainfall regime, with a long rainy season starting from March to May and a short one from mid-October to December. The mean annual temperature ranges between 18 and 23°C [20] and mean annual rainfall between 1500 and 2000 mm at 1500 m asl [21]. During the coolest period (June to August), the average temperature ranges between 18 and 20°C. In this area, agricultural landscape is dominated by agroforestry systems known as Chagga home gardens, where coffee is grown in small farms with banana and other food crops as well as fruit trees used as shade trees, such as *Persea americana* Mill. and *Mangifera indica* L. [21].

### Field surveys

The antestia bug, *A*. *thunbergii* was identified as one of the two main pests of coffee in the studied area. The second major pest was the African coffee white stem borer, *Monochamus leuconotus* Pascoe (Coleoptera: Cerambycidae) [22]. The antestia bugs observed on the transect were identified as the subspecies *A*. *thunbergii bechuana* Kirkaldy, using the morphological identification key and distribution maps proposed by Greathead [2]. This subspecies differs from *A*. *thunbergii ghesquierei* Carayon, which has a slightly different coloration and a more western distribution in east Africa [1]. The two other species of antestia bugs found on coffee in east Africa, *A*. *intricata* and *A*. *facetoides* were not found on the transect. Four field surveys were conducted spanning across three different climatic seasons, two during the cool dry season in June 2014 and 2015, one at the beginning of the short rainy season in October 2014 and one during the warm dry season in January 2015. *Antestiopsis thunbergii* populations were assessed in 24 coffee farms, selected in groups of 4 at approximately 1100, 1200, 1300, 1400, 1500 and 1600 m asl (Fig 1). The selected farms were representative of the area; they were usually small, with about 100 coffee trees, and the age of the trees was between 10 and 50 years. The farms were homogeneously planted with the same traditional Arabic coffee variety, originated from breeding of Bourbon-type and Kent-type old varieties. In each farm, 15 trees were randomly selected on both sides of a diagonal across the farm. Individuals of *A*. *thunbergii* were counted visually on the selected trees, irrespective of the development stage of the pest. Presence of eggs was noted, without counting.

### Temperature data

Real-time air temperature was recorded hourly throughout the year 2013 as an activity of the CHIESA project (http://chiesa.icipe.org/) using climate data loggers (iButtons Hygrochron, Maxim Integrated, San Jose, USA). This data was then borrowed to the project for analyses presented in the present paper. Nine data loggers were deployed across the transect (Fig 1). Data loggers were hanged on tree branches at approximately 2 m above the ground to record air temperature under shade. Data logger geographical position and elevation were recorded using a handheld Global Positioning System (GPS).

For 2055 temperature estimates, we used simulated temperature dataset (AFRICLIM version 3.0) of the Representative Concentration Pathway scenario 4.5 (RCP 4.5) of the fifth assessment report of Intergovernmental Panel on Climate Change (IPCC-AR5). RCP 4.5 emission scenario was selected because it is a moderate scenario, which includes long term global emissions of greenhouse gases, land use and land cover in a global economic framework [23]. The simulated temperature data were downscaled to a resolution of 30″ (1 km) using General Circulation Models (GCMs) and Regional Climate Models (RCMs), with WorldClim grids as baseline. Data are well documented by Platts et al. [24] and freely accessible at http://www.york.ac.uk/environment/research/kite/resources/. We downloaded the temperature dataset for the year 2055 as raster layers in tiff format. Then, we used point sampling tool in QGIS software (version 2.18.4) to extract temperature data from layers, using the geographical position of data loggers on the transect as coordinates.

### Statistical analysis of the survey data

The relationship between *A*. *thunbergii* populations and elevation was determined through a regression analysis, using a generalised linear model with a Poisson distribution. R programming environment [25] was used for all calculations. The dependent variable was the total number of bugs per farm, i.e. the total number of bugs counted on 15 coffee trees. This variable was regressed against the elevation variable. The same regression analysis was computed for the 4 observation times. Poisson distribution assumes that the mean and variance of the count data are equal, and over-dispersion occurs when the variance is greater than the mean. This case was detected in our data and was corrected using “dispmod” package in R [26]. With this method, the goodness of fit of Poisson regression model is assessed on the basis of the deviance (log-likelihood ratio statistic), which has a distribution approximating to chi-squared, and is represented as χ^2^. To display the current distribution of the bug on the study transect, we mapped the bug density (mean number of bugs per tree) using the regression model obtained for each observation time, and elevation data computed from the digital elevation model (DEM) of the transect. Calculation and mapping were done using the “raster” package in R programming environment [27]. To analyse the effect of season on *A*. *thunbergii* populations, generalised linear model with a Poisson distribution was fitted to the total number of bugs per plot as dependent variable and season as independent variable. Once a significant difference was detected, data were submitted to post hoc analysis for mean comparison, using Tukey test at *P* = 0.05.

### Prediction of distribution based on temperature-dependent models

#### Demographic parameters’ simulation and model validation

To predict the distribution of *A*. *thunbergii* populations as impacted by elevation, we used the temperature-dependent development models reported in the study by Azrag et al. [11]. The laboratory colonies used in this study were initiated with individuals of the same subspecies, *A*. *thunbergii bechuana*, collected in the same area than this targeted in the present study. Models were developed using life table data collected at 7 constant temperatures (18, 20, 23, 25, 28, 30 and 32°C). Mathematical functions were fitted to immature stage development time and rate, mortality rate, female fecundity and adult senescence, using the software Insect Life Cycle Modelling (ILCYM, version 3.0) [19]. Using these models, *A*. *thunbergii* life table parameters were simulated for different constant temperatures, *viz*. 1) the gross reproductive rate (*GRR*), which is defined as the average number of daughters produced by a living female throughout her life time, 2) the net reproductive rate (*R*_*o*_), which is the rate of multiplication per generation taking into account the mortality rate of immature stages, 3) the mean generation time (*T*) which is defined as the mean time (in days) between the birth of parents and the birth of offspring, 4) the doubling time (*Dt*), which is the time (in days) that is required for the population to double, 5) the intrinsic rate of increase (*r*_*m*_), which is defined as the innate capacity of a population to grow, and 6) the finite rate of increase (*λ*), which is the average per capita multiplication factor per one time unit [11, 19, 28].

For the present study, an additional experiment was conducted at fluctuating temperature in order to validate the models developed by Azrag et al. [11] at constant temperatures. The validation experiment was carried out in an open insectary from April to October 2015 at International Centre of Insect Physiology and Ecology (*icipe*), Kenya. A total of 154 individuals of *A*. *thunbergii* were reared from egg to adult using coffee green berries and leaves as diet (see [13] for a complete description of the rearing method). During the experiment, temperature was recorded every hour using a data logger (iButtons Hygrochron, Maxim Integrated, San Jose, USA). The daily mean temperature ranged between 17.4 and 28.1°C, while the daily minimum temperature ranged between 10.6 and 18.7°C, and the daily maximum temperature between 20.6 and 36.9°C. *Antestiopsis thunbergii* demographic parameters were calculated from the models using fluctuating temperature data. Then, the calculated parameters were compared to the values simulated from data collected at constant temperatures [11]. Consistency between simulated and calculated values of demographic parameters was good enough to validate the models and undertake risk index calculation.

#### Calculation and mapping of infestation risk indices

*Antestiopsis thunbergii* distribution as impacted by elevation was displayed using the following three indices, i) the establishment risk index (ERI), ii) the generation index (GI) and iii) the activity index (AI). The ERI gives the capacity of an insect to establish in a particular area based on temperature. When mapped, the ERI enables the visualisation of the geographical areas suitable for the insect’s establishment and survival. The index is 1 when all immature stages of the pest survive throughout the year in the particular area. Otherwise, the number of days in which a single stage would not survive (100% mortality) are counted and divided by 365 (number of Julian days), and then subtracted from 1. The GI estimates the mean number of generations that an insect may produce within a year, and it is estimated by the number of Julian days (365) divided by the estimated generation time (*T*). The AI assesses the potential distribution and abundance of the species; the AI is closely related to the finite rate of population increase (*λ*) (population growth rate), which takes into account the whole life history of the pest. It is calculated by taking the log of the products of estimated finite rates of increase calculated for each Julian day. For example, an AI value of 4 gives a potential population increase factor of 10 000 in one year. These indices were computed and mapped on the same transect using “*index interpolator*” in ILCYM [19] based on i) the temperature-dependent development models and related demographic parameters reported by Azrag et al. [11], ii) the digital elevation model (DEM) for the study area (elevation was used as a co-variable in ASCII format), and iii) the current and future temperature data (daily minimum and maximum temperatures). Indices were initially calculated for each geographical point where temperature data were recorded or simulated, and then interpolated on the surface of the digital elevation model using thin plate algorithm interpolation method [19].

In order to better characterise the changes in the pest’s distribution between current and future temperatures, we randomly extracted risk index values from a total of 160 geographical points in groups of 20, located at every 100-m elevation across the transect, using point sampling tool in QGIS. The same geographical points were used to extract indices for current and future temperatures. Then, extracted data were tested for normality and a Wilcoxon test was applied to the data in R (version 3.3.0) [25] to compare the mean of the indices in current and future temperature conditions at each elevation.

## Results

### Effect of elevation and season on *A*. *thunbergii* populations

**Fig 2:**
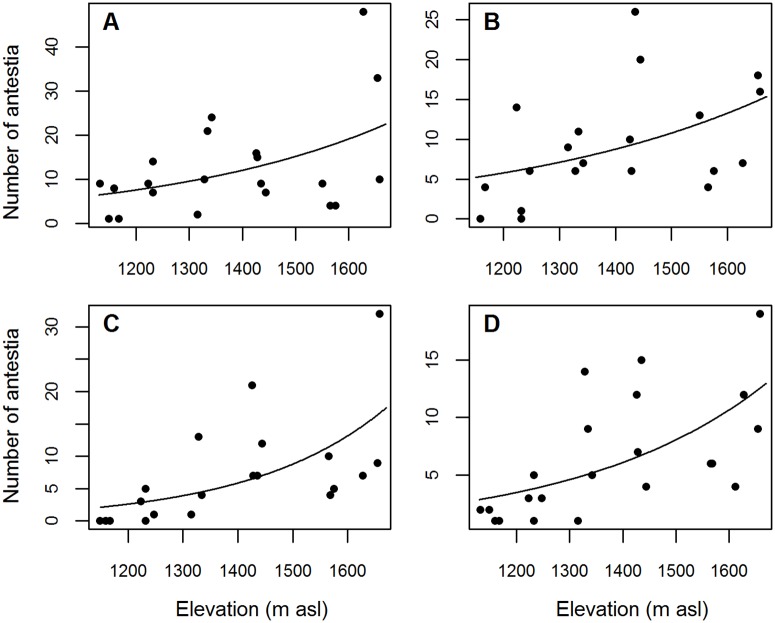
Observed *Antestiopsis thunbergii* populations (total number of bugs on 15 coffee trees) in relation to elevation on Kilimanjaro transect for the different evaluation periods. (A) June 2014, cool dry season, (B) October 2014, short rainy season, (C) January 2015, warm dry season, (D) June 2015, cool dry season.

**Fig 3:**
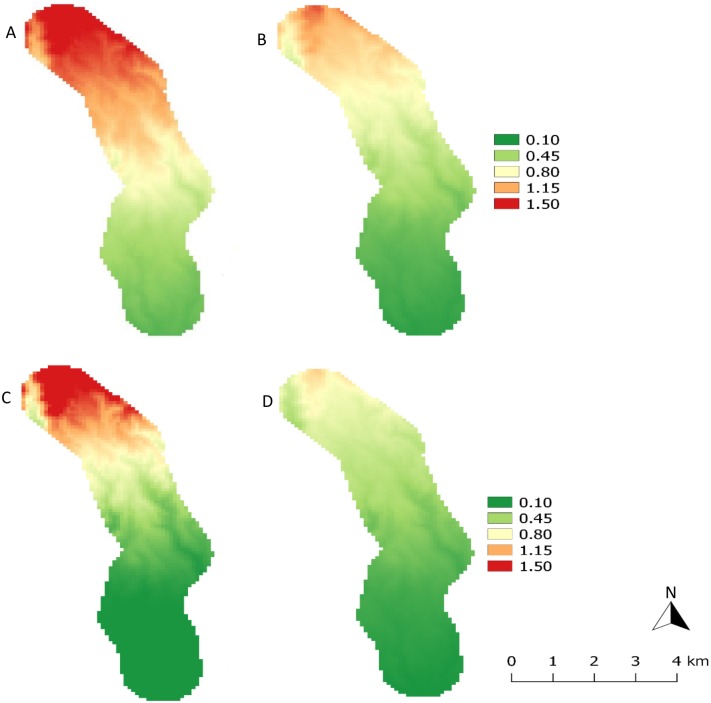
Distribution of *Antestiopsis thunbergii* populations (mean density per tree) over the elevation transect on Kilimanjaro, for the different evaluation periods. (A) June 2014, cool dry season, (B) October 2014, short rainy season, (C) January 2015, warm dry season, (D) June 2015, cool dry season.

The impact of elevation on the total number of *A*. *thunbergii* per farm (total counts on 15 trees) was significant, with an increase in populations together with elevation for the four different evaluation periods (June 2014 (cool dry season): χ^2^ = 20.159, *df* = 19, z = 2.553; *P* < 0.05; June 2015 (cool dry season): χ^2^ = 20.848, *df* = 22, z = 2.877; *P* < 0.01; October 2014 (short rainy season): χ^2^ = 19.065, *df* = 18, z = 2.302, *P* < 0.05; January 2015 (warm dry season): χ^2^ = 16.868, *df* = 18, z = 3.622, *P* < 0.0001) (Fig 2). Total number of *A*. *thunbergii* per farm was very variable even for farms located at similar elevation and for the same evaluation period, especially for the highest elevations. For example, in June 2014, populations ranged between 4 and 48 bugs for 15 trees for elevation around 1600 m asl. For the same elevation, in January 2015, population ranged between 5 and 32 bugs for 15 trees (Fig 2). Maps of Fig 3 confirm that population densities were the highest at the top of the transect (1600–1700 m asl), whatever the period. They exceeded 1.5 bug tree^-1^ at the top in June 2014 and January 2015, but were lower in October 2014 and June 2015, with about 1 bug tree^-1^. At the bottom of the transect (1000–1100 m asl), densities were around 0.1 bug tree^-1^ and were slightly higher in June 2014, when compared to the other periods. In the cold season, populations of *A*. *thunbergii* reached 12.5 bugs per farm, which was significantly higher, compared to the warm dry season (6.6 bugs per farm) (χ^2^ = 63.2, *df* = 68, *P* < 0.05).

### Risk indices under current temperature

**Fig 4:**
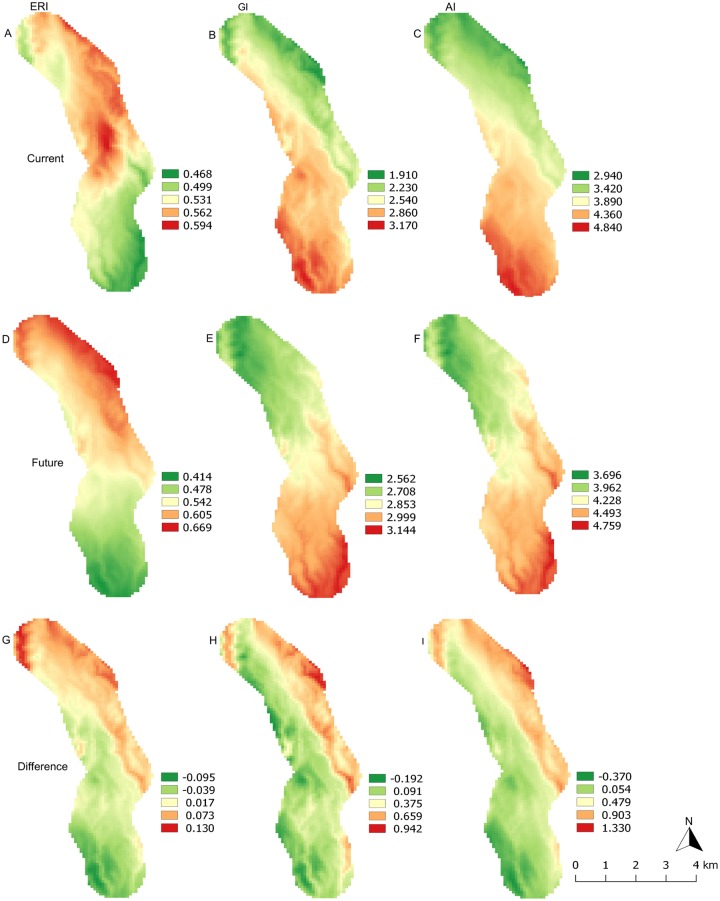
Distribution of establishment risk index (ERI), generation index (GI) and activity index (AI) of *A*. *thunbergii* on Kilimanjaro elevation transect calculated from the temperature dependent-development models under current (2013) and future (2055) temperature conditions. (A) ERI, (B) GI and (C) AI under current temperature; (D) ERI, (E) GI and (F) AI under future temperature projections; (G) ERI, (H) GI and (I) AI absolute difference between future and current temperature conditions.

Mean current temperature measured with data loggers varied in the range of 18–23°C from the top to the bottom of the transect. Maps of risk index distribution show that the establishment risk index is clearly linked to elevation with the lowest values (ERI ≈ 0.47) at the bottom of the transect and the highest values (ERI ≈ 0.59) at the highest elevations (Fig 4A). By contrast, the generation index is higher in the lower zone of the transect (GI ≈ 3.17) when compared to the top (GI ≈1.91) (Fig 4B). The activity index follows a similar trend with AI ≈ 4.84 at the bottom and AI ≈ 2.94 at the top of the transect (Fig 4C).

### Change in risk indices under future temperature

**Fig 5:**
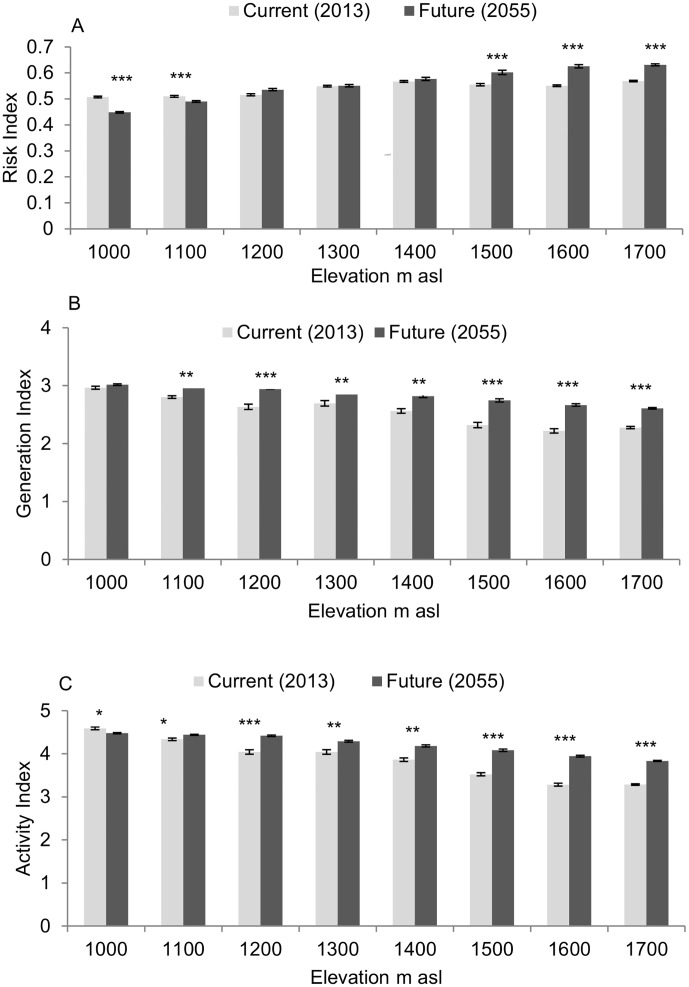
Change in risk indices for *A*. *thunbergii* populations of Kilimanjaro transect between current (2013) and future (2055) temperature conditions, plotted against elevations. (A) Establishment risk index (ERI), (B) generation index (GI), (C) activity index (AI). * = *P* < 0.05, ** = *P* < 0.001, *** = *P* < 0.0001.

Minimum temperature predicted for 2055 will be higher than temperature we measured in 2013 across the transect by 0.1–0.3°C, whereas maximum temperature predicted for 2055 will be higher by 0.6–1.8°C (Table 1). Change in temperature varies with elevation but there is no clear pattern in the variations. Under future temperatures, the ERI will change across the transect with difference ranging between ˗0.09 and 0.13 at the bottom and top of the transect, respectively (Fig 4D and 4G). The ERI will significantly decrease at elevations between 1000 and 1100 m asl (1000 m asl: *W* = 210, *P* < 0.0001; 1200 m asl: *W* = 206, *P* < 0.0001), it will remain unchanged between 1200 and 1400 m asl, and will significantly increase between 1500 and 1700 m asl (*W* = 210, *P* < 0.0001 for 1500, 1600 and 1700 m asl) (Fig 5A). The GI will change with difference between ˗0.19 and 0.94 from the bottom to the top of the transect (Fig 4E and 4H). It will remain unchanged at elevation around 1000 m asl (*W* = 138, *P* = 0.1089) and will significantly increase for elevations between 1100 and 1700 m asl (1100 m asl: *W* = 202, *P =* 0.0001; 1200 m asl: *W* = 210, *P* < 0.0001; 1300 m asl: *W* = 171, *P* = 0.0068; 1400 m asl: *W* = 203, *P* = 0.0001; 1500 m asl: *W* = 209, *P* < 0.0001; 1600 and 1700 m asl: *W* = 210, *P* < 0.0001) (Fig 5B). The AI will change in a range of ˗0.37–1.33 from the bottom to the top of the transect (Fig 4F and 4I). The AI will decrease at elevations of 1000 and 1100 m asl (1000 m asl: *W* = 162, *P* = 0.0166; 1100 m asl: *W* = 160, *P* = 0.0200) and will increase significantly for elevations between 1200 and 1700 m asl (1200 m asl: *W* = 201, *P* < 0.0001; 1300 m asl: *W* = 169, *P* = 0.0084; 1400 m asl: *W* = 203, *P* = 0.0001; 1500 m asl: *W* = 210, *P* < 0.0001; 1600 m asl: *W* = 210, *P* < 0.0001; 1700 m asl: *W* = 210, *P* < 0.0001) (Fig 5C).

**Table 1:** Change in minimum and maximum temperatures (mean ± SD) between current (2013) and future (2055) climatic conditions on selected locations along the Kilimanjaro transect. The current temperatures were recorded using iButtons Hygrochron data loggers in the selected locations and future temperatures obtained from AFRICLIM 3.0 climatic projections of RCP 4.5 scenario.

Elevation (m)	Coordinate (°)		Minimum temperatures (°C)			Maximum temperatures (°C)		
	Longitude	Latitude	Current	Future	Difference	Current	Future	Difference
1081	37.461845	-3.357280	17.18±1.29	17.28±1.23	0.10±0.74	28.59±3.47	30.42±2.56	1.83±1.20
1249	37.474743	-3.341794	16.67±1.31	16.78±1.18	0.11±0.28	29.53±3.72	29.63±2.39	0.10±1.43
1419	37.469891	-3.327607	15.31±1.48	15.58±1.14	0.27±0.52	27.41±2.78	27.98±2.38	0.57±0.84
1533	37.465210	-3.312971	14.60±1.44	14.94±1.29	0.34±0.42	26.58±4.20	27.34±2.32	0.76±2.91
1705	37.456239	-3.286082	13.85±1.44	14.02±1.34	0.17±0.56	25.58±3.29	26.34±2.14	0.76±1.50

## Discussion

### Impact of elevation on population density from field observations

In the last decades, generalized linear models (GLMs) have been widely used in ecological studies because of their ability to deal with different error structures associated with occurrence data [29]. Also, they are more flexible and suit better to analysing ecological relationships such as the relationship between insect distribution and elevation [29]. In our study Poisson regression well predicted the relationship between *A*. *thunbergii* populations and elevation. Our results showed that *A*. *thunbergii* was present at all elevations of the study area with the highest population densities at the highest elevations, whatever the climatic seasons. Our results confirm those of previous studies reporting that *A*. *thunbergii* is present in coffee at elevations between 1000 and 2100 m asl, with a preference for the highest elevations, especially for the subspecies *A*. *thunbergii bechuana* [1, 2]. In our study, a mean density of 1 to 1.5 bug tree^-1^ was reported for elevation between 1600 and 1700 m asl across the different climatic seasons. In most countries of east Africa, this density is considered as an economic threshold beyond which an intervention for controlling the pest is required [4, 8, 9]. Maximum density we obtained was a bit more than 3 bugs tree^-1^ at around 1630 m asl. This is rather low when compared to maximal densities of 45 bugs tree^-1^ obtained for the subspecies *A*. *thunbergii ghesquierei* in Rwanda by Foucart and Brion [30]. Evaluation method used in that study was the “pyrethrum tests”, a knock-down technique based on pyrethrum spraying. In our study, visual counts on coffee trees may have led to underestimated densities, since the bugs tend to hide between berries or on the underside of the leaves. However, our results also suggest that densities of *A*. *thunbergii* could have been higher on the transect at elevation above 1700 m asl if coffee had been present.

In our study, high variation in *A*. *thunbergii* density was observed even between farms located at similar elevations. This indicates that, besides elevation, other crucial factors were involved in the infestation level. Some ecological traits of *A*. *thunbergii* suggest that the bug prefers cool environments; populations are usually more abundant in bushy coffee trees and in shaded plantations, especially at medium and low elevation [9, 31]. Our study area is characterised by agrosystems known as Chagga home gardens, where vegetation usually develops in four layers: big trees for fruits, wood and shade, banana trees, coffee trees, and maize and/or vegetables at ground level [21]. Such complex systems lead to a wide range of shade and microclimate conditions, which may explain the variation we obtained in the bug density for similar elevations.

Our results also showed that *A*. *thunbergii* populations varied between seasons, with the highest populations in June, during the coolest period of the year. During our field surveys, additional observations revealed that all the development stages, from eggs to adults were present on trees during this period, whereas eggs were not observed during the warmer seasons. These results are consistent with report from Van der Meulen and Schoeman [32], who recorded higher populations in the cold season, for the same species in South Africa. Authors suggested that in addition to lower temperatures, coffee fruiting cycle might have been involved in the bug seasonal variations. More specifically, the presence of green berries on trees might have favoured immature stage survival and development. This explanation may be valid in our study since coffee trees bore developing green berries in June, whereas from October to December, berries ripened and were harvested (Azrag AGA, personal communication).

### Modelling infestation risk under current temperature

Several bioclimatic models such as BIOCLIM, CLIMEX and MaxEnt [33, 34, 35] have been adapted for predicting the distribution and abundance of insect species, based on field and/or laboratory data [16, 36, 37, 38]. These models use occurrence points of the species to predict, based on bioclimatic variables, its distribution over a broad geographic area (for example, at global, continental or regional scale). However, in our study, we used ILCYM software to predict the distribution of *A*. *thunbergii* at a local scale, namely over an elevation transect of a few dozen square kilometres. ILCYM software uses a deductive approach of modelling, incorporating the detailed knowledge of the pest life history from laboratory experiment to predict where the species can occur. Also, the software is able to predict the pest distribution at a very small geographical scale, which provides efficient information for risk mitigation strategies that need to be implemented at local scale. Nevertheless, the main limitation of ILCYM is that the software uses only temperature variable to predict the pest distribution and does not incorporate other climatic variables such as rainfall and relative humidity. Future versions of the software should consider such variables to improve prediction accuracy.

The first index, the ERI, characterises the area suitable for the survival and establishment of an insect. The ERI is computed based on survival of immature stages and has a range of 0–1. In our study, the ERI for current temperature ranged from 0.47 to 0.59. This result suggests that *A*. *thunbergii* immature stages are able to survive all over the transect for half the year, at least. This result also indicates that the bug distribution may not be limited to elevation range of the transect. In fact, the relationship we observed between the ERI and elevation suggests that areas above 1700 m may be even more favourable for *A*. *thunbergii* establishment than the areas covered by the transect. Such assertion is supported by previous studies reporting that *A*. *thunbergii* is more common in Arabica coffee at the highest elevations that can reach 2100 m asl in east Africa [2].

In the study by Azrag et al. [11], the temperature-dependent mortality models showed that the constant temperature range of 22–24°C was optimal for the survival of all *A*. *thunbergii* immature stages. This range matches the mean annual temperatures for the elevations between 1000 and 1300 m asl, where the ERI was the lowest in our study. This contradictory result is linked to the fact that the ERI calculation uses daily minimum and maximum temperatures. Thus, extreme daily maximum temperatures observed at the lowest elevations of the transect may be lethal for the bug immature stages, leading to low ERI. This is supported by the study by Ahmed et al. [13], who recorded a mortality rate of 89% at 30°C for immature stages of the bug.

The second index, the GI, estimates the mean number of generations year^-1^. The GI is computed based on the generation time (*T*) and does not take survival into account. In our study, the GI decrease with increasing elevation is linked to the relationship between insect development time and temperature. For temperatures allowing survival of at least some individuals, an increase in temperature invariably shortens insect development, as demonstrated by Azrag et al. [11] at constant temperatures for *A*. *thunbergii* in a temperature range of 18–32°C. In our study, GI ranged 1.9–3.2 generations year^-1^ from the top to the bottom of the transect. When maintained at constant temperature, the bug was able to have 2.6–4.4 generations year^-1^ under temperature in range 19–25°C [11]. The field study by Van der Meulen and Schoeman [31] reported up to 4 overlapping generations year^-1^ in South Africa. However, our results suggest that in our study area, it is unlikely that *A*. *thunbergiii* populations would be able to reach 4 generations year^-1^ considering the low risk of establishement at low elevation.

The third index, the AI, indicates the potential population growth and is an indicator of the pest severity and spread risk. The AI is computed based on the finite rate of increase (*λ*), which is the exponential of the intrinsic rate of increase (*r*_*m*_). As such, the AI takes the whole life cycle of the pest into account. Our predictions revealed that the AI distribution was very similar to that of the GI with a decrease in values from the top to the bottom of the transect. This can be explained by the results obtained by Azrag et al. [11] for simulated life table parameters. In this study, intrinsic rate of increase and generation time varied in an opposite trend with an increase of temperature in the range 18–23°C. This range matches with temperature range of the transect. It is then consistent that the AI and the GI showed similar distribution on the transect. Also, results from Azrag et al. [11] suggest that the AI reached its highest value in the bottom of the transect and should have decreased for lower elevations, contrary to the GI. In our study, the AI ranged 2.94–4.84 from the top to the bottom of the transect. According to AI formula [28], these values mean that the bug population may potentially grow by a factor of ≈ 870 at the top and ≈ 69,200 at the bottom of the transect, if temperature was the only factor involved in population variation.

### Comparison between risk indices and current distribution of the pest

In this study, the ERI and the pest populations had similar distribution and a close relationship to elevation on the transect. By contrast, the GI and the AI indicate an opposite relationship with elevation. The ERI is therefore the best index to predict *A*. *thunbergii* distribution at a local scale. Similar result has been reported in similar studies conducted on other pests at world scale, like the potato tuber moth *Phthorimaea operculella* (Zeller) [28] or fruit flies *Ceratitis rosa* Karsch and *Ceratitis quilicii* De Meyer [39]. In addition, our results suggest that immature stage survival is a crucial parameter of the pest distribution. Immature stage susceptibility to high temperature that may occur during the hottest hours of the day may be one of the main factors preventing the pest from establishing in plantations at low elevation. This is supported by the fact that *A*. *thunbergii* adopts behaviours aiming at avoiding highest temperatures: the pest is usually more active in the morning and in the evening, and avoids direct sunlight during the day by hiding in the leaf cover or between berries [1]. Susceptibility to desiccation, which is also linked to sunlight exposure, cannot be ignored, and more studies are needed to understand the impact of moisture on antestia bug development.

In our study, *A*. *thunbergii* was found all over the transect for the 3 different seasons. Clearly populations were small in the bottom of the transect, but some individuals were recorded around 1200 m asl even during the warm season which is not favourable. This suggests a permanent establishment of the bug throughout the year on the transect. A permanent establishment should match with an ERI close to 1. However, in our study, the ERI ranged 0.47–0.59. This contradictory result has already been reported in other studies for other pests at world scale, where the ERI value for permanent establishment was either > 0.6 [39] or > 0.8 [28]. In our study, it seems that an ERI > 0.5 is enough to illustrate a permanent establishment of *A*. *thunbergii*. As already mentioned above, highly diversified coffee systems in our study area lead to a large variety of microclimates and it is likely that the bug finds favourable temperature conditions even at the lowest elevations. Another disputable result in our study is the mismatch between the high AI values we obtained, illustrating high potential growth rates for the bug populations and low densities globally observed in coffee. The issue of the counting method has already been mentioned but factors other than temperature were undoubtedly involved in the limitation of the bug population. The most important limiting factor for antestia bugs is probably the pressure of natural enemies, especially egg parasitoids. Most field surveys reported egg parasitism rates in the range 40–95% and rates exceeding 80% were not rare [1]. An interesting study may be to assess the impact of temperature on egg parasitoid development.

### Change in risk indices with temperature increase

Our predictions showed that by the year 2055, *A*. *thunbergii* will still be present in Arabica coffee plantations along the study area. However, the infestation risk will change with rising temperature. Unsurprisingly, at low elevation an increase in temperature will lead to a decrease in the ERI. This decrease is estimated at 4–12% at elevations between 1000 and 1100 m asl. A more surprising result in our study is the ERI increase at the top of the transect, which is estimated at 8–14% for elevation between 1500 and 1700 m asl. One of the reasons may be the reduction of periods of the year with low temperatures lethal for *A*. *thunbergii* immature stages. Azrag et al. [11] recorded high mortality rates for the bug nymphs, especially between the 2^nd^ and 5^th^ stages, for constant temperatures between 16 and 18°C. However, this result should be considered with caution since current and future temperatures are not measured the same way in our study.

As mentioned above, the GI reflects the impact of temperature on insect development time and does not take mortality into account. It is therefore not surprising that the GI is expected to increase all over the transect with rising temperature. By contrast, the AI is expected to decrease at the bottom of the transect. This result confirms our assumption that, for current temperature, the AI reached its highest value at the lowest elevations. An increase in temperature may therefore lead to a shift of the AI highest values from low to higher elevation.

Globally, our results suggest that an increase in the risk of infestation by *A*. *thunbergii* is expected for coffee at high elevation. Several studies showed that rising temperatures will highly influence coffee production in eastern Africa. For example, in Tanzania, suitable areas for coffee growing are expected to shift from 1000–2000 m asl to 1300–2300 m asl due to temperature increase of 1.9–2.6°C [21, 40, 41, 42]. Therefore, a reduction in antestia bug infestation at low elevations as demonstrated in our study will have limited consequences for coffee production in the future. By contrast, our results suggest that *A*. *thunbergii* will be able to follow the shift in coffee production area, and will threaten renowned Arabica coffee at the highest elevations, as it does today.

### Conclusions and recommendations

In conclusion, the temperature-based models and related risk indices we used in this study allowed the prediction of *A*. *thunbergii* distribution as impacted by elevation. Immature stage susceptibility to extreme temperatures has proven to be a crucial factor limiting population growth. Other biophysical factors, such as shade, farmer practices, moisture, coffee phenology or natural enemies are undoubtedly involved in the pest population dynamics. These factors could explain the high variation in *A*. *thunbergii* populations we obtained from field observations. Therefore, we recommend further field studies to elucidate the relationships between these factors and *A*. *thunbergii* population dynamics. Such understanding would help refine predicting models for this major pest of coffee.

In the future, infestation by the bug will remain high at high elevation and the bug will be able to follow the shift of coffee areas to higher elevations. Coffee farmers of east Africa highlands have to be ready for that. Our results strengthen one of the current recommendations for the control of antestia bug: a good pruning of the coffee bushes. Good pruning practices enhance producing branches exposure to sun and improve productivity. In addition, these practices are detrimental for antestia bug development because they expose the pest to extreme temperature conditions, which can be lethal, especially for immature stages. Shading coffee is a valid recommendation to mitigate temperature increase and control some pests that prefer warmer conditions, like the coffee berry borer. For antestia bug, shade management recommendations should take elevation into account, since they can have opposite effect on pest infestation depending on the global climate of the target area.
